# Association of the ACE2 rs879922 Polymorphism with Glaucoma Risk and Serum ACE2 Levels

**DOI:** 10.3390/jcm15166168

**Published:** 2026-08-08

**Authors:** Anna Cieślińska, Natalia Krzykowska, Dominika Rozmus, Ewa Fiedorowicz, Patrycja Kuklo, Janusz Płomiński, Andrzej Grzybowski

**Affiliations:** 1 Department of Biochemistry, Faculty of Biology and Biotechnology, University of Warmia and Mazury in Olsztyn, Oczapowskiego 1A, 10-719 Olsztyn, Poland; 2 Department of Ophthalmology, University of Warmia and Mazury in Olsztyn, Warszawska 30, 10-082 Olsztyn, Poland; 3 Orthopaedic and Trauma Teaching Hospital, 05-400 Otwock, Poland; 4 Institute for Research in Ophthalmology, Foundation for Ophthalmology Development, Gorczyczewskiego 2/3, 61-553 Poznan, Poland

**Keywords:** angiotensin, angiotensin II converting enzyme, polymorphism, SNP, ACE2, rs879922

## Abstract

**Background/Objectives:** Glaucoma is a progressive optic neuropathy and one of the leading causes of irreversible blindness worldwide. Despite the important role of elevated intraocular pressure in disease development, genetic factors such as polymorphisms in the angiotensin-converting enzyme 2 (ACE2) gene may contribute to disease susceptibility. This study aims to evaluate the correlation between the rs879922 polymorphism in the ACE2 gene and glaucoma risk, as well as to assess its relationship with serum ACE2 protein levels. **Methods:** Patients diagnosed with glaucoma and healthy individuals were enrolled in this study. The rs879922 polymorphism was tested using the PCR-RFLP method. Serum ACE2 concentrations were measured using the ELISA method and expressed as mean ± standard deviation. **Results:** The rs879922 polymorphism was significantly associated with glaucoma in the allelic (OR = 2.04, *p* = 0.01) and recessive (OR = 2.63, *p* = 0.01) models, but not in the dominant model (OR = 1.80, *p* = 0.21). Genotype distributions deviated from Hardy–Weinberg equilibrium in both groups. Mean serum ACE2 levels were higher in glaucoma patients in comparison to controls (5.23 vs. 3.29 ng/mL). Genotype-stratified analysis revealed that in controls, the GG genotype was associated with the highest ACE2 levels, whereas in glaucoma patients, the CC genotype showed the highest concentrations. **Conclusions:** The rs879922 polymorphism in the ACE2 gene may be associated with glaucoma susceptibility and influences serum ACE2 levels. The relationship between genotype and ACE2 concentration appears to differ between glaucoma patients and healthy individuals, suggesting a potential interaction between genetic variation and disease status.

## 1. Introduction

**Figure 1:**
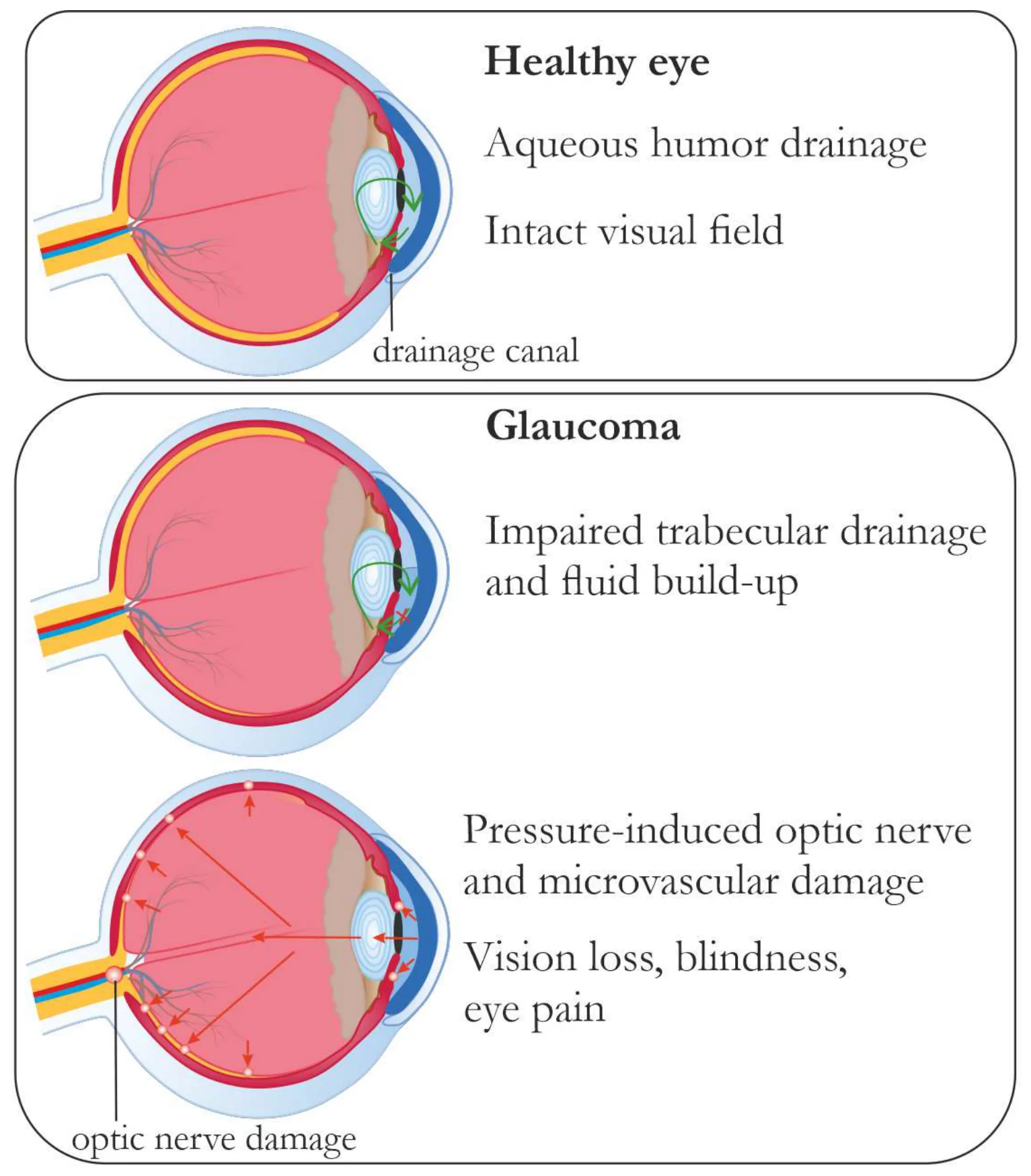
Scheme of glaucoma.

Glaucoma is a multifactorial optic nerve disease associated with gradual degeneration of retinal ganglion cells (RGCs), and their axons, resulting in structural changes in the optic nerve head and corresponding loss of visual function [1]. Although elevated intraocular pressure (IOP) is a major risk factor and therapeutic target, glaucoma can also develop at “normal” IOP, suggesting the involvement of additional mechanical, vascular, and genetic factors. More than 70 million people worldwide are affected by glaucoma [2,3], making it a major contributor to irreversible vision loss. The distribution of primary open-angle glaucoma (POAG) and primary angle-closure glaucoma (PACG) varies according to demographic and environmental factors, including age, ethnicity, geographic region, and socioeconomic status [4,5]. Because the disease is often asymptomatic in its early stages, many patients remain undiagnosed until significant visual field loss has occurred (Figure 1).

Recent studies showed that the renin–angiotensin system (RAS) may be involved in glaucoma pathophysiology. Beyond its classical systemic role in regulating blood pressure and vascular tone, a local ocular RAS has been detected in tissues such as the trabecular meshwork, retina, ciliary body, or choroid [6]. Elevated angiotensin II (Ang II) levels have been detected in the aqueous humor and trabecular meshwork of glaucoma patients, where Ang II promotes extracellular matrix remodeling, oxidative stress, and fibrotic changes that impair aqueous humor outflow and elevate IOP [7]. Moreover, Ang II signaling has been associated with neuroinflammation and RGC degeneration in animal models, indicating that RAS dysregulation may contribute to both pressure-dependent and pressure-independent mechanisms of glaucomatous optic neuropathy [8]. By contrast, the ACE2–Ang-(1–7)–Mas receptor axis exhibits protective biological effects, including vasodilation, reduced fibrosis, and neuroprotection, and has been linked to decreased IOP [9,10]. These findings raise the possibility that pharmacological modulation of RAS could offer novel therapeutic strategies in glaucoma. ACE2 is a critical enzyme of the protective ACE2–Ang-(1–7)–Mas axis within the RAS. It converts Ang II into Ang-(1–7), thereby opposing Ang II-mediated vasoconstriction, oxidative stress, fibrosis, and inflammation. ACE2 expression has been demonstrated in multiple ocular structures, including the cornea, retina, ciliary body, and retinal pigment epithelium [11,12]. Experimental studies indicate that ACE2 protects retinal tissue by attenuating inflammation, oxidative stress, and apoptosis, whereas reduced ACE2 expression promotes retinopathies, especially in the context of diabetes and hypertension. In addition to its protective ocular effects, ACE2 has gained attention as the cellular receptor utilized by SARS-CoV-2, providing a potential explanation for ocular manifestations of COVID-19 and viral damage to eye tissue [13,14].

Genetic variants within ACE2 may modulate its expression or activity, thereby influencing susceptibility to ocular and systemic diseases. One such variant is rs879922, a single nucleotide polymorphism (SNP) located in a non-coding intronic region of the ACE2 gene (chrX:15572684, GRCh38.p14). This polymorphism involves a cytosine (C)-to-guanine (G) substitution and has been suggested to influence gene regulation, splicing or expression despite not altering the amino acid sequence of the encoded protein [15,16]. Several studies suggest that rs879922 is linked to variability in ACE2 mRNA and protein levels across human tissues, likely through effects on regulatory elements or mRNA stability [11,12]. Functional assays further indicate that rs879922 variants can modulate promoter activity and post-transcriptional processes, highlighting its potential role in disease mechanisms.

Despite evidence from animal studies, the relevance of ACE2 modulation in human glaucoma remains insufficiently understood. Determining how ACE2 expression varies in human ocular tissues and how genetic variations such as rs879922 influence ACE2 protein levels may provide new insights into glaucoma pathogenesis and identify novel therapeutic targets.

## 2. Materials and Methods

### 2.1. Study Population

Between 2019 and 2024, individuals were enrolled in this case–control study at the Voivodal Specialistic Hospital in Olsztyn, Poland. Biological specimens and clinical information were collected as part of the study procedures. Clinical data were retrieved from medical documentation and participant questionnaires. All subjects provided written informed consent before participation. Ethical approval was granted by the local Bioethics Commission, and the study was performed in accordance with the Declaration of Helsinki (protocol codes 49/2019–25 April 2019 and 5/2022–20 January 2022).

The study population was categorized into two groups:

(1) Glaucoma group (G): The glaucoma group included patients with a confirmed diagnosis of glaucoma established at least one year before enrollment who had been receiving topical antiglaucoma treatment for more than one year. The type of topical intraocular pressure-lowering medication was not considered an exclusion criterion. In our center, first-line glaucoma management consists of topical medications, whereas surgical treatment is considered only when medical therapy is insufficient. Therefore, patients with newly diagnosed glaucoma or elevated intraocular pressure diagnosed less than one year before enrollment were not eligible for inclusion. Patients with a history of any glaucoma surgery or laser glaucoma procedure, regardless of when or where the procedure had been performed, were excluded from the study. In contrast, previous cataract surgery and ongoing topical glaucoma treatment did not constitute exclusion criteria. Additional exclusion criteria included age below 18 years, lack of written informed consent, the presence of systemic comorbidities other than diabetes, and coexistence of glaucoma and diabetes in the same patient. To ensure clearly defined study groups, patients in the glaucoma group did not have diabetes, whereas patients in the diabetes group did not have glaucoma. Patients with a history of major intraocular surgeries other than cataract surgery were also excluded.
(2) Control group was classified as healthy based on the absence of ocular and systemic diseases and underwent a standard ophthalmological examination to confirm that they did not have glaucoma. All volunteers met the following conditions: no history of glaucoma or intraocular pressure-lowering treatment, normal intraocular pressure measured during the ophthalmic examination, normal optic nerve appearance on slit-lamp biomicroscopy and/or fundus examination, no visual-field defects suggestive of glaucoma (if perimetry was performed), no history of major ocular surgery, and no significant systemic comorbidities.

The study sample was selected to ensure well-defined clinical characteristics. The demographic characteristics of the study population, including age and sex, are presented in Table 1.

### 2.2. Sample Collection and DNA Isolation

Blood and serum samples (2.0 mL each) were obtained from 169 study participants. All samples were promptly transferred to the laboratory for immediate analysis or storage at −20 °C until further processing. Genomic DNA was extracted from peripheral blood using the Blood Mini DNA Isolation Kit and Sherlock AX (A&A Biotechnology, Gdańsk, Poland), followed by purification according to the manufacturer’s protocol. Extracted DNA was adjusted to a final concentration of 20 ng/µL and stored at −20 °C until use. Serum samples unsuitable for testing due to compromised quality were excluded from analysis.

### 2.3. ACE2 rs879922 Genotype Analysis

PCR reactions were carried out in a final volume of 17.5 µL containing DreamTaq™ Green Master Mix (Thermo Scientific, Waltham, MA, USA), specific primers (F: 5′-TCTCTTTCTCCAGACATGAACTC-3′; R: 5′-AAGATCTTGTCCCCGTCATC-3′; Sigma-Aldrich, Burlington, MA, USA) designed using Primer3Plus (version 3.3.0), genomic DNA template, and nuclease-free water (Thermo Scientific, Waltham, MA, USA). Reactions were carried out in a Mastercycler Nexus thermal cycler (Eppendorf, Hamburg, Germany). PCR was performed using an initial denaturation at 95 °C for 5 min, followed by 35 cycles of denaturation (95 °C, 60 s), annealing (66 °C, 60 s), and extension (72 °C, 60 s). A final extension step at 72 °C for 10 min was included before cooling the samples to 4 °C. The expected PCR product (377 bp) was verified by electrophoresis on 1.5% agarose gel (Promega, Fitchburg, WI, USA) stained with GelGreen Nucleic Acid Gel Stain (Biotium, Fremont, CA, USA). Genotyping was performed by restriction fragment length polymorphism (RFLP) using a FastDigest BfaI (FspBI) restriction enzyme (Thermo Scientific, Waltham, MA, USA), according to the manufacturer’s instructions. The digestion products were separated on 2.5% agarose gel and visualized as follows: GG genotype—377 bp; CG genotype—377, 234, and 143 bp; CC genotype—234 and 143 bp.

### 2.4. Determination of Serum ACE2 Protein Concentrations

Serum ACE2 concentrations were determined in duplicate using a commercially available Human ACE2 ELISA Kit (cat. No. E3169Hu, BT Lab Bioassay Technology Laboratory, Shanghai, China) according to the manufacturer’s protocol. All assay components were allowed to reach room temperature prior to use. The required number of strips was placed in the assay frame, while unused strips were stored at 2–8 °C. Standards (50 µL) were added to the designated wells, while 40 µL of each sample was added to sample wells followed by 10 µL of biotinylated anti-human ACE2 antibody. Streptavidin-HRP solution (50 µL) was then added to both standards and sample wells (excluding blank controls). After incubation (60 min at 37 °C), and washing steps (5 times), substrate solutions A and B (50 µL each) were added to each well and the plates were incubated in the dark (10 min at 37 °C). The reaction was terminated with 50 µL of stop solution, and absorbance was measured at 450 nm using a microplate reader.

### 2.5. Statistical Analysis

The relative association between allelic/genetic groups was assessed by calculating odds ratios (ORs) with 95% confidence intervals and *p*-values using Fisher’s exact test implemented in Python (version 3.13) with the SciPy library. For each comparison, 2 × 2 contingency tables were constructed for the presence or absence of specific genotypes in the Control and Ophthalmology patient groups. Hardy–Weinberg equilibrium (HWE) was tested for each group separately using chi-square tests based on observed and expected genotype counts, calculated in Python. Heterozygotes were treated as CG regardless of the allele order (i.e., GC was counted as CG).

ACE2 concentrations were converted to numerical values and presented as means ± standard deviations (SD). Statistically significant differences were marked on figures with *, **, *** and **** corresponding to *p* < 0.05, *p* < 0.01, *p* < 0.001, and *p* < 0.0001, respectively.

Generative AI-assisted technology was used for language editing and improvement of readability.

## 3. Results

### 3.1. Allele and Genotype Distribution

**Figure 2:**
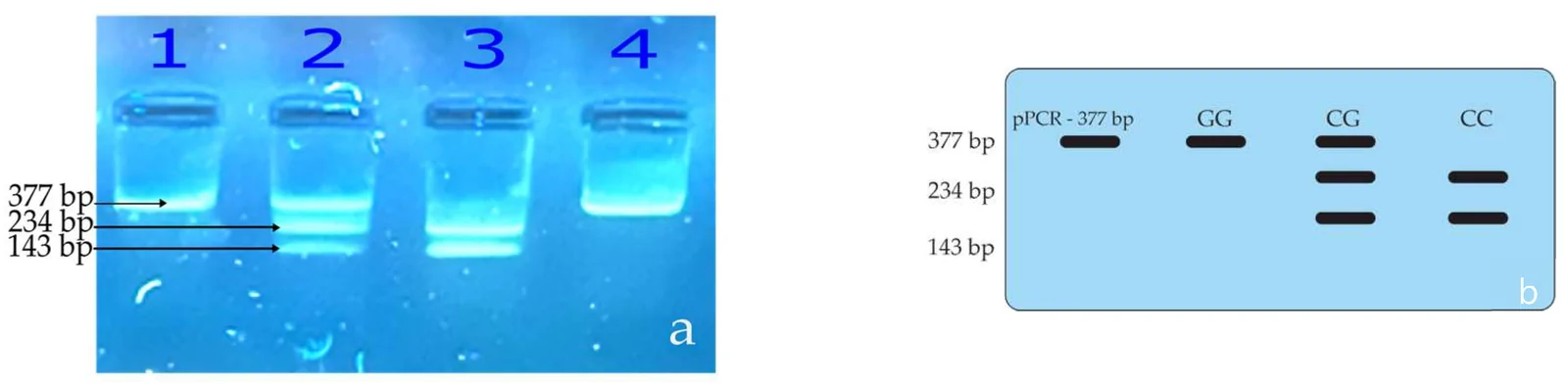
Representative (**a**) and theoretical (**b**) electropherogram of enzymatic digestion products on agarose gel after electrophoresis. The visible paths show: path 1 dominant homozygote GG—1 bar (377 bp), path 2 heterozygote CG—three bars (377 bp, 234 bp, 143 bp), path 3 recessive homozygote CC—two bars (234 bp, 143 bp), path 4 PCR product (377 bp).

The observed genotype distribution of the rs879922 ACE2 polymorphism deviated from Hardy–Weinberg equilibrium in both the control group (χ^2^ = 24.03, *p* < 0.0001) and the glaucoma group (χ^2^ = 7.07, *p* = 0.0078). Representative electropherogram results are shown in Figure 2.

Detailed genotype counts, allele frequencies, and the associations between rs879922 and glaucoma are summarized in Table 2.

### 3.2. ACE2 Levels and Correlations

In the whole population (controls + glaucoma patients), the mean serum ACE2 protein concentration was 3.68 ng/mL. The analysis showed that the glaucoma group had a higher mean concentration (5.23 ng/mL) compared with the control group (3.29 ng/mL). When analyzed by genotype at the rs879922 polymorphic site, clear differences were observed between genotypes. In the control group, individuals with the GG genotype demonstrated the highest mean of ACE2 serum levels (4.05 ng/mL), while the CC genotype mean was lower (1.97 ng/mL) and the CG genotype showed the lowest mean concentration (1.79 ng/mL). In the glaucoma group, the CC genotype had the highest mean of ACE2 levels (8.79 ng/mL), followed by CG mean (4.67 ng/mL) and GG mean (3.42 ng/mL). These findings indicate that the influence of genotype on ACE2 levels differs between healthy controls and glaucoma patients. While in controls, GG homozygotes show the highest ACE2 level (maximum of 13.4 ng/mL), in glaucoma patients CC homozygotes exhibit the highest maximum ACE2 level (19.79 ng/mL). The results of ACE2 concentrations are presented in Table 3 below.

**Figure 3:**
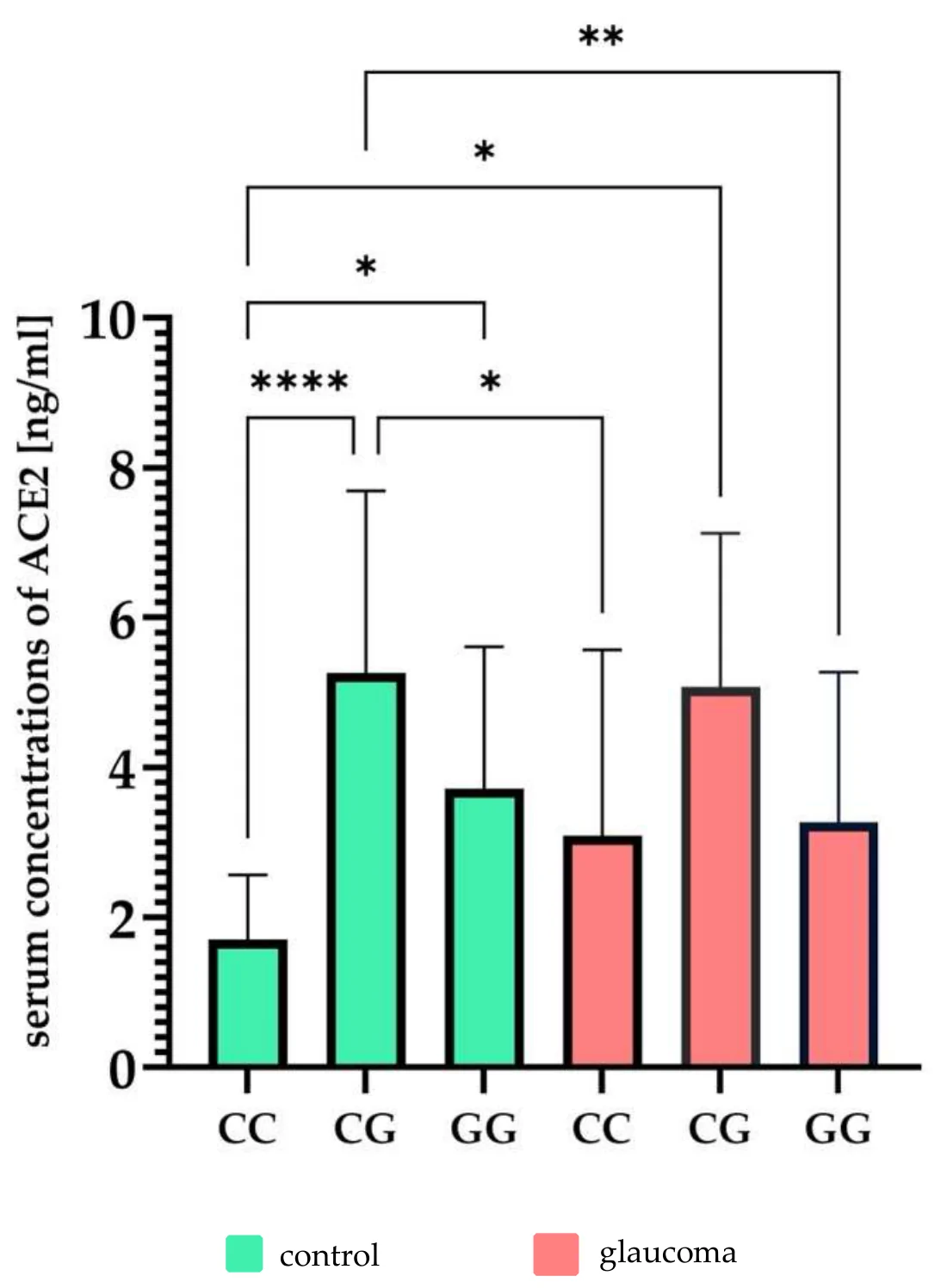
Serum ACE2 concentrations according to ACE2 rs879922 genotypes in glaucoma patients and controls. Statistical significance was denoted as follows: * *p* < 0.05; **, *p* < 0.01 and ****, *p* < 0.0001.

The comparison between the groups is shown in Figure 3.

**Figure 4:**
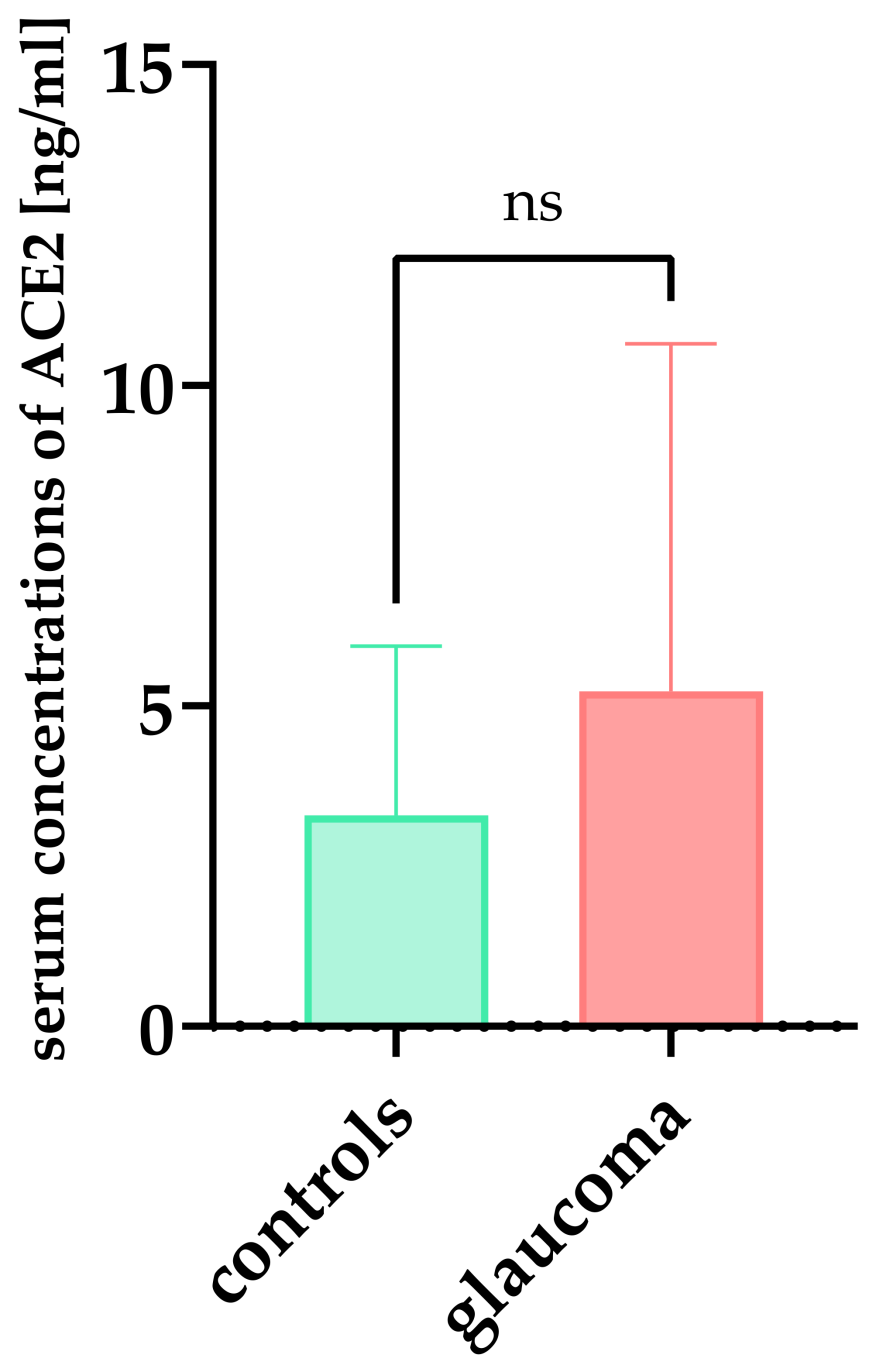
Serum ACE2 concentrations in glaucoma patients and controls. Plot presents means with SD. Statistical significance was denoted as follows: ns, not significant (*p* ≥ 0.05).

A comparison between mean of glaucoma and control group is presented in Figure 4. Serum ACE2 concentrations were higher in glaucoma patients compared with controls. However, this difference did not reach statistical significance (Mann–Whitney U test, *p* = 0.296). Thus, no significant difference in circulating ACE2 levels was observed between the two groups.

This may indicate a potential interaction between genotype and disease status. Overall, these findings highlight genotype-dependent variability in ACE2 serum concentrations and suggest that the relationship between genotype and ACE2 levels may be modified by glaucoma status.

## 4. Discussion

The CC genotype was more frequent among glaucoma patients and conferred more than a twofold increase in disease risk, consistent with the recessive model (Table 2). The CG genotype was more frequent among glaucoma patients relative to healthy controls. However, it was not associated with consistently elevated ACE2 levels. Serum ACE2 concentrations were highest in the CC genotype among glaucoma patients and in the GG genotype among controls, indicating that genotype-dependent effects on ACE2 expression differ between groups.

While the heterozygous CG genotype was relatively common in glaucoma patients, it did not show a clear pattern of increased ACE2 expression, suggesting a more complex, non-linear relationship between genotype and protein levels. These findings indicate that genotype-dependent variation in ACE2 expression exists, but its pattern differs between glaucoma patients and controls, pointing to a possible interaction between genetic background and disease status in the regulation of ACE2 levels.

The genetic aspect of our findings is consistent with prior studies in other populations. Some studies [17,18,19] linked rs879922 to RAAS activation, hypertension, dyslipidemia, and type 2 diabetes. Carriers of the C allele or CC genotype had higher levels of renin, ACE, and angiotensins I/II and greater cardiovascular risk. Other studies associated rs879922 with left ventricular hypertrophy [15,20] and metabolic abnormalities, including obesity and lipid imbalance in adolescents [21]. Overall, these findings suggest that rs879922 is a pleiotropic variant affecting multiple organ systems through the dysregulation of RAAS balance. Our data extend this concept to ocular disease, suggesting that ACE2 genetic variation may contribute to glaucoma susceptibility, potentially via effects on microcirculation, oxidative stress, and tissue remodeling in the retina and trabecular meshwork.

In our study, serum ACE2 concentration appeared to be associated primarily with the rs879922 genotype rather than with disease status alone. A significant relationship between ACE2 levels and the study group was observed only after stratification by rs879922 genotype (Figure 3). Although mean ACE2 levels were higher in glaucoma patients than in controls (Figure 4), this difference did not reach statistical significance, suggesting that genetic background may contribute more strongly to ACE2 variability than the presence of glaucoma itself. This suggests that systemic ACE2 dysregulation may accompany glaucoma and could reflect compensatory activation of the ACE2–Ang-(1–7)–Mas pathway in response to chronic RAS imbalance. Although the difference between groups was modest, the trend is consistent with evidence from systemic diseases in which elevated soluble ACE2 has been interpreted as a marker of underlying vascular stress and RAS overactivation. Previous research has confirmed the presence of ACE2 in ocular tissues and aqueous humor (AH). Holappa et al. [10] reported median ACE2 concentrations of ~2.3 ng/mL in AH but found no significant case–control differences. Similarly, Ren et al. [22] detected ACE2 in AH of POAG patients and controls, again with no significant differences. These data support the concept of a local ocular renin–angiotensin system (RAS), in which ACE2 counteracts Ang II-driven fibrosis, oxidative stress, and inflammation, but do not show consistent alterations in ACE2 levels between glaucoma and non-glaucoma eyes. To date, no studies have directly compared serum ACE2 levels between glaucoma patients and controls, making our work original.

Our findings also parallel observations from systemic diseases. In hypertension, diabetes, and cardiovascular disorders, circulating soluble ACE2 (sACE2) is often elevated, typically interpreted as a compensatory response to Ang II overload. However, increased sACE2 in serum does not necessarily indicate increased tissue ACE2 activity, as shedding of membrane ACE2 by ADAM17 may reduce its local availability. In glaucoma, a similar mechanism could apply. Serum ACE2 may be modestly elevated, while ocular ACE2 activity remains insufficient, favoring Ang II–AT1R–mediated pro-fibrotic and neurodegenerative effects.

A review of published studies shows that ACE2 has been measured in a variety of systemic and ocular conditions, although the results remain heterogeneous across diseases. In COVID-19, serum soluble ACE2 (sACE2) levels were detectable (~3.2–3.5 ng/mL), with some reports of higher levels in severe cases, although differences between SARS-CoV-2-positive and -negative individuals were not always significant. In chronic kidney disease and hemodialysis, median sACE2 concentrations were markedly lower (≈0.16 ng/mL), and higher baseline values were associated with increased risk of infection-related hospitalization. In diabetes, patients exhibited significantly reduced plasma ACE2 compared to healthy controls, with additional decreases linked to hypoglycemic therapy. In cardiovascular, viral, and pulmonary diseases, a meta-analysis reported consistently elevated ACE2 expression, interpreted as compensatory activation of the ACE2–Ang-(1–7) axis (Table 4).

Our studies confirm that ACE2 is readily measurable in human serum and plasma and that its levels vary widely depending on disease context. However, no consistent glaucoma-specific serum/plasma ELISA data have been reported to date. Our results therefore fill an important gap by providing the first direct serum-based ACE2 measurements in glaucoma patients. Together, these findings underscore the variability of ACE2 regulation across conditions and highlight the novelty of our study, which provides the first direct data on serum ACE2 levels in glaucoma. Our study has several limitations. First, the sample size was modest, and findings require replication in larger and ethnically diverse cohorts. Second, serum ACE2 may not fully reflect ocular ACE2 activity due to variable shedding and systemic influences. Third, we could not directly assess ACE2 activity or local ocular concentrations in aqueous humor or retinal tissue.

The observed association between the rs879922 polymorphism and glaucoma susceptibility may have potential implications for future risk stratification and personalized therapeutic approaches. As ACE2 is a key regulator of the renin–angiotensin system, genetic variability may influence individual susceptibility to glaucoma and response to treatments targeting RAS-related pathways. However, these potential clinical applications require validation in larger cohorts and functional studies before ACE2 genotyping can be considered a useful biomarker in clinical practice.

## 5. Conclusions

Our data indicate that rs879922 polymorphism in the ACE2 gene may influence glaucoma susceptibility and serum ACE2 levels. The CC genotype was linked to a higher risk of glaucoma, according to the recessive model, while genotype-dependent differences in ACE2 concentrations were observed between glaucoma patients and controls. Notably, ACE2 levels were highest in CC carriers among glaucoma patients and in GG carriers among controls, suggesting that the relationship between genotype and ACE2 expression is modified by disease status. These findings support the role of ACE2 genetic variation in modulating systemic RAS balance and its potential contribution to glaucoma pathogenesis. Further studies are warranted to elucidate the interplay between systemic and ocular ACE2 activity in glaucoma.

## Figures and Tables

**Table 1:** Characteristics of research groups.

Demographic Data	Control (n = 80)	Glaucoma (n = 57)	*p*-Value
Age (years mean (SD))	69.5 (8.3)	73.4 (7.5)	0.52
Sex: n (%)			
Female:	39 (49)	39 (70)	0.34
	χ^2^ = 5		

**Table 2:** Association between the ACE2 rs879922 polymorphism and glaucoma risk based on allelic, dominant, and recessive genetic models.

Genotype	Control [n (%)]	Glaucoma [n (%)]	Control + Glaucoma [n (%)]	
GG	52 (65)	24 (42)	76 (55)	
CG	14 (18)	18 (32)	32 (23)	
CC	14 (18)	15 (26)	29 (21)	
Alleles frequency (F)				
F_G_	0.74 (74)	0.58 (58)	0.67 (67)	
F_C_	0.26 (26)	0.42 (42)	0.32 (32)	
Model	**OR**	**95% CI Lower**	**95% CI Upper**	***p*** **-Value**
Allelic (G vs. C)	2.04	1.22	3.41	0.01
Dominant (GG + CG vs. CC)	1.80	0.80	4.06	0.21
Recessive (GG vs. CG + CC)	2.63	1.31	5.28	0.01

**Table 3:** ACE2 concentrations in the glaucoma and control groups according to rs879922 genotype.

	Control			Glaucoma			All Controls	All Glaucoma
Rs879922 Genotype	GG	CC	CG	GG	CC	CG	-	-
Number of values	52	14	14	24	15	18	80	57
Minimum [ng/mL]	1.0	0.85	0.71	0.3	0.87	0.97	0.71	0.3
Maximum [ng/mL]	13.4	6.76	5.12	16.32	19.78	16.66	13	20
Range [ng/mL]	12.4	5.91	4.41	16.02	18.91	15.69	13	19
Mean [ng/mL]	4.05	1.97	1.79	3.42	8.79	4.67	3.3	5.2
Std. Deviation [ng/mL]	2.81	1.82	1.137	3.849	6.81	4.74	2.6	5.4
Std. Error of Mean [ng/mL]	0.39	0.49	0.3039	0.79	1.76	1.12	0.29	0.72
Lower 95% CI of mean [ng/mL]	3.27	0.915	1.132	1.796	5.02	2.32	2.7	3.8
Upper 95% CI of mean [ng/mL]	4.83	3.02	2.445	5.047	12.56	7.03	3.9	6.7

**Table 4:** ACE2 levels (measured using ELISA test) in various diseases.

Disease	Research Group	Results	Reference
COVID-19 (SARS-CoV-2 infection)	N = 94/SARS-CoV-2 patients vs. N = 94 SARS-CoV-2–negative patients	sACE2 detectable (~3.27 ± 0.41 ng/mL in positives vs. ~3.56 ± 0.44 ng/mL in negatives); difference not statistically significant	[23]
Hypertension + COVID-19	N = 200/patients in four different antihypertensive medication classes	sACE2 and Ang II levels varied depending on therapy	[24]
Chronic Kidney Disease/Hemodialysis	N = 724/clinically stable patients on maintenance hemodialysis	Median sACE2 ≈ 0.16 ng/mL. higher baseline sACE2 linked to a higher risk of hospitalization due to infections	[25]
Diabetes	N = 174/diabetes mellitus patients, n = 230 Controls	Diabetics with chronic disease showed lower plasma ACE2 (≈2974 ± 2197 pg/mL) vs. healthy controls (≈4308 ± 2353 pg/mL). Treatment with glucose-lowering medications was linked to reduced circulating ACE2 levels	[26]

## Data Availability

The datasets generated and/or analyzed during the current study are not publicly available due to ethical and privacy restrictions related to patient data. Aggregated data are available from the corresponding author upon reasonable request.

## Abbreviations

The following abbreviations are used in this manuscript:

Abbreviation	
ACE2	Angiotensin-converting enzyme 2
ADAM17	A disintegrin and metalloproteinase 17
AH	Aqueous humor
Ang II	Angiotensin II
Ang-(1–7)	Angiotensin-(1–7)
CI	Confidence interval
COVID-19	Coronavirus disease 2019
GC	Heterozygous genotype (G/C)
GG	Homozygous genotype (G/G)
CC	Homozygous genotype (C/C)
HWE	Hardy–Weinberg equilibrium
ng/mL	Nanograms per milliliter
OR	Odds ratio
PACG	Primary angle-closure glaucoma
POAG	Primary open-angle glaucoma
RAAS	Renin–angiotensin–aldosterone system
RAS	Renin–angiotensin system
RGCs	Retinal ganglion cells
sACE2	Soluble angiotensin-converting enzyme 2
